# Acupuncture for treating chronic stable angina pectoris-associated anxiety and depression: A protocol for systematic review and meta-analysis

**DOI:** 10.1097/MD.0000000000021583

**Published:** 2020-07-31

**Authors:** Mingqi Tu, Yongliang Jiang, Jie Yu, Binjun Liao, Jianqiao Fang

**Affiliations:** a Department of Neurobiology and Acupuncture Research, the Third Clinical Medical College, Zhejiang Chinese Medical University, Key Laboratory of Acupuncture and Neurology of Zhejiang Province; b Department of Acupuncture and Massage, Affiliated Hangzhou First People's Hospital, Zhejiang University School of Medicine, Hangzhou, China.

**Keywords:** acupuncture, anxiety, chronic stable angina pectoris, depression, meta-analysis

## Abstract

**Background:**

There are numerous studies worldwide on the use of acupuncture as complementary therapy for chronic stable angina pectoris (CSAP). However, the high morbidity of CSAP-associated anxiety and depression is often overlooked. This protocol of systematic review and meta-analysis aims to assess whether acupuncture is effective as a complementary therapy for anxiety and depression in patients with CSAP.

**Methods:**

The following 8 databases will be searched from inception to February 2020 with no language restrictions: PubMed, Excerpt Medical Database, Web of Science, the Cochrane Library, Chinese Biomedical Database, China National Knowledge Infrastructure, VIP Database and Wanfang Database. Eligible randomized controlled trials and controlled clinical trials will be included. Study selection, data extraction, and risk of bias assessment will be performed independently by 2 reviewers, differences will be resolved by the third reviewer. The primary outcomes include the level of anxiety or depression measured by qualified scales, angina attack frequency, and angina pain intensity. Revman 5.3 software will be used to perform the assessment of the risk of bias and data synthesis. The review will grade the quality of the evidence based on the Grading of Recommendation, Assessment, Development, and Evaluation system.

**Results:**

This systematic review and meta-analysis will provide reliable evidence about the effect and safety of acupuncture as a complementary therapy for CSAP-associated anxiety and depression.

**Conclusion:**

The conclusion of this study will be published in a peer-reviewed journal.

**Ethics and dissemination:**

This review will not involve private information of participants, so the ethical approval will not be required. The results will be disseminated in a peer-reviewed journal or conference presentation. Important protocol modifications will be updated on PROSPERO.

**PROSPERO registration number:**

CRD42020165492.

## 1 Introduction

Chronic stable angina pectoris (CSAP) is a clinical syndrome caused by temporary reversible myocardium ischemia and hypoxia. Exercise, emotion, or other stress can usually trigger angina attack that may also occur spontaneously. The main symptom is paroxysmal colic, obtuse pain, or press sensation of pain in prothorax near sternum, may radiate to the back or upper limb.^[1]^ CSAP is a prevalent heart disease and is a leading cause of death worldwide. Anxiety and depression are common complications in patients with CSAP.^[2–5]^ However, the high prevalence of CSAP-related anxiety or depression is often being ignored. Previous researches indicate that more than 40% patients with CSAP have complication of anxiety and depression, which make the prognosis worse.^[3,6]^ Moreover, anxiety and depression can further aggravate angina attack.^[7–9]^ Clinically, CSAP, anxiety, and depression are often treated independently. Anti-ischemic drugs, revascularization, lifestyle modifications, and control of risk factors are primary treatments for CSAP, while anxiety and depression can be treated by drugs and psychotherapy.^[1,10,11]^ Although these treatments have satisfactory curative effects, they have some relative risks and side effects.^[12–14]^ At present, there is no treatment that can reduce angina symptoms and suppressing anxiety and depression at the same time.

Many previous studies have evaluated the efficacy of acupuncture in the treatment of CSAP.^[3,15–17]^ Most of them indicate it has a positive effect. Some studies also proved that acupuncture is effective for treating anxiety and depression.^[18–20]^ However, the effectiveness and safety of acupuncture for CSAP with anxiety and depression remain unclear. Therefore, we evaluated it through systematic review and meta-analysis.

## 2 Methods

### 2.1 Study design and registration

Preferred Reporting Items for Systematic Reviews and Meta-Analysis (PRISMA) will be carried out in this systematic review and meta-analysis. The study was registered in PROSPERO (https://www.crd.york.ac.uk/prospero/display_record.php?RecordID=165492).

### 2.2 Eligibility criteria

#### 2.2.1 Types of studies

The review will include randomized controlled trials and controlled clinical trials. Two arm or 3-arm parallel designed trials will be included. Case report, cross-sectional studies, comments, cohort studies, animal experiments, and reviews will be excluded.

#### 2.2.2 Types of participants

Trials including participants who meet the diagnostic criteria of CSAP will be included. There are no restrictions on age, sex, or race of adult patients. The study does not require participants to be diagnosed with anxiety or depression. Because the focus of the study is on evaluating the change of anxiety or depression level after acupuncture, it means that an assessment of anxiety or depression is required in the trials.

Trials including participants who meet any of the following conditions will be excluded: pregnancy or lactation; other severe diseases not effectively controlled (such as severe heart failure, severe arrhythmias, severe psychiatric).

#### 2.2.3 Types of interventions

The intervention must be described as acupuncture. Acupuncture, acupuncture combined with Chinese medicine, acupuncture combined with conventional drugs will be included. However, other interventions without needle insertion will be excluded (e.g., moxibustion only, acupoint application, massage, or transcutaneous electrical nerve stimulation). Stimulation methods include manual acupuncture and electro-acupuncture.

#### 2.2.4 Types of comparator(s)/control

If the control group of the trial is sham acupuncture, standard/routine care, or conventional drugs, the trial will be included. Trials will be excluded if they are designed to compare different acupuncture techniques or acupoint-selection, or compared acupuncture with other complementary or alternative therapeutics.

#### 2.2.5 Types of outcome measures

##### 2.2.5.1 The primary outcomes

1. The level of anxiety or depression measured by qualified scales, such as the Hamilton Anxiety or Depression Scale and the Zung Self-Rating Anxiety or Depression Scale;
2. Angina attack frequency;
3. Angina pain intensity.

If data are available, we will analyze them at baseline, posttreatment, and follow-up(s).

##### 2.2.5.2 The secondary outcome

Adverse events.

### 2.3 Search strategy

#### 2.3.1 Electronic searches

Two independent researchers (MT and JY) will search the following electronic databases from inception to February, 2020, which includes PubMed, Excerpt Medical Database, Web of Science, Chinese Biomedical Database, China National Knowledge Infrastructure, VIP Database, and Wanfang Database. No language restrictions will be applied.

The following Medical Search Headings (MeSH) will be used to search in PubMed and other English databases: angina, angina pectoris, stable angina pectoris, angor pectoris, chronic stable angina pectoris, stenocardia, stenocardias, acupuncture, eletro-acupuncture, acupuncture-moxibustion, needle, needling, depression, depressions, depressive, anxiety, anxieties, nervousness. The search strategy for PubMed is shown in Table 1. In Chinese database, Chinese characters with the same meaning will be used for literature retrieval.

**Table 1:**
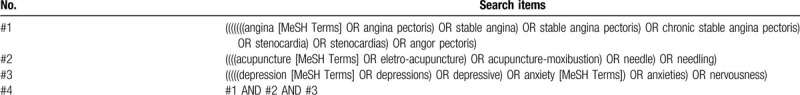
Search strategy in PubMed.

#### 2.3.2 Searching other resources

We search ongoing trials with unpublished data in Chinese clinical registry (http://www.chictr.org.cn/) and the NIH clinical registry-clinicaltrials. gov (https://www.clinicaltrials.gov/). Relevant systematic reviews of all potential publications will be manually retrieved and reviewed to further locate additional trials. Incomplete data will be obtained by contacting the corresponding authors if possible.

### 2.4 Data collection and analysis

#### 2.4.1 Selection of studies

**Figure 1:**
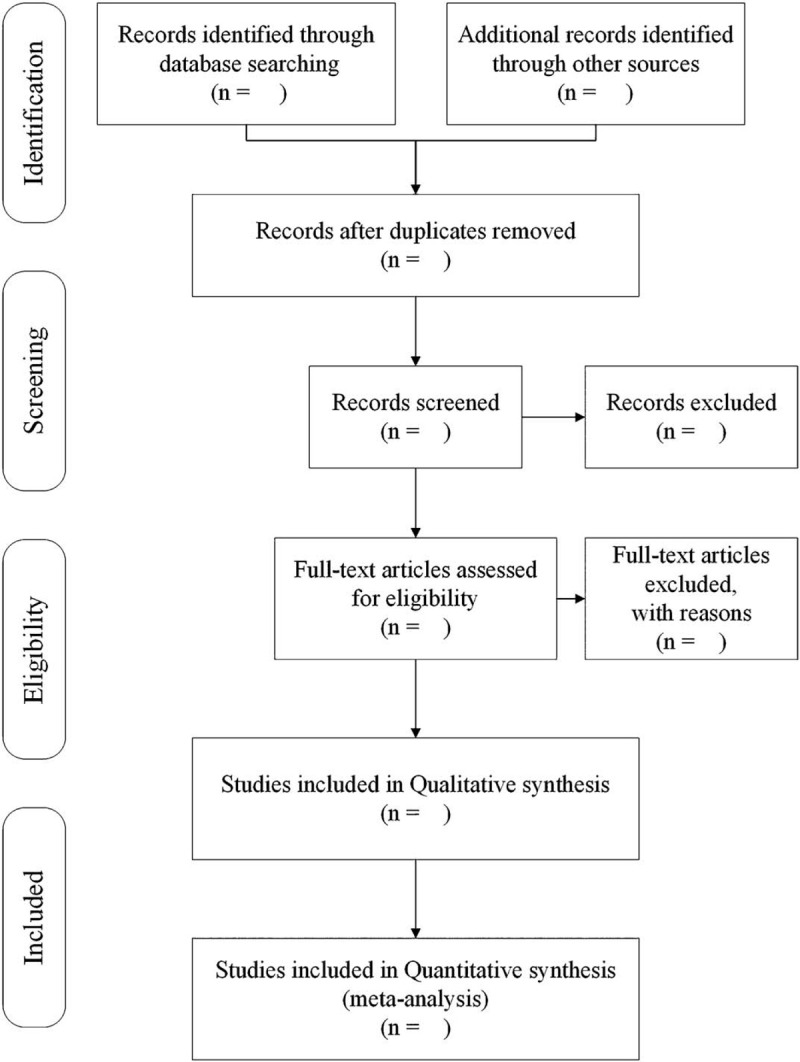
Study selection flow diagram.

Two reviewers (MT and JY) will screen the titles and abstracts of all included studies independently to find potentially eligible studies. When studies could not be eliminated by reading the titles and abstracts, researchers will read the full text independently to decide whether it should be excluded. Any disagreements in the process will be resolved by discussion or arbitrated by a third reviewer (JF). The reasons for exclusion will be recorded in detail. The procedure of study selection is shown in Fig. 1.

#### 2.4.2 Data extraction and management

After filtering the final eligible studies, the data will be extracted independently by 2 reviewers. The following data will be extracted: first author's name, year of publication, number of participants, age, sex, duration of disease, duration of follow-up periods, details of acupuncture (such as stimulation methods, sessions, frequency); control group, outcomes. If relevant data is missing, we will contact the corresponding author. Any disagreement in the above process will be resolved by discussion between the 2 reviewers and further disagreements will be arbitrated by the third reviewer (JF).

#### 2.4.3 Assessment of risk of bias

Two reviewers (MT and JY) will assess the risk of bias of eligible studies independently according to the Cochrane Risk of Bias Assessment Tool. Risk of bias includes 7 domains: random sequence generation, allocation concealment, blinding of participants and personnel, blinding of outcome assessment, incomplete outcome data, selective reporting, and other bias. We will evaluate the included trials and classify them into 3 levels: high risk, low risk, and unclear. Disagreements will be resolved by discussion with the third reviewer (JF).

#### 2.4.4 Measures of treatment effect

Weighted mean difference or standardized mean difference with its 95% confidence interval (CI) will be used for continuous data and risk ratio with its 95% CI will be used for dichotomous data.

#### 2.4.5 Dealing with missing data

We will contact the corresponding author or relevant author by reviewers (MT and JY) to obtain the missing data. If we cannot obtain missing data, we will exclude these studies.

#### 2.4.6 Data synthesis and assessment of heterogeneity

Data synthesis will be completed with clinical data by using RevMan software (V.5.3). The χ^2^ test or I^2^ test will be used in the forest plot to assess the quantify impact of the statistical heterogeneity. If the *P* value in χ^2^ test is <.10 or I^2^ is >50%, the heterogeneity across studies will be statistically significant and the random-effects model will be used. Otherwise, we will choose a fixed-effect model. If there is considerable heterogeneity, we will carry out subgroup analysis to identify the sources of heterogeneity.

#### 2.4.7 Assessment of reporting biases

If necessary, we will choose appropriate methods (such as the Funnel plots, the Egger test, and the Begg test) to analyze potential publication bias.

#### 2.4.8 Subgroup analysis

If data are available, researchers will conduct subgroup analyses according to variations in characteristics of trial participants and acupuncture treatments. Planned subgroup analyses will be performed in terms of various factors, such as age, duration of disease, different stimulation methods of acupuncture, control group, and different assessment scales.

#### 2.4.9 Sensitivity analysis

If necessary, sensitivity analysis will be performed to monitor the robustness of the primary decision made in the review process. Several decision nodes will be considered, such as methodological weaknesses, sample size, and missing data.

### 2.5 Assessment method of evidence quality

We will grade the quality of the evidence based on the Grades Profiler as the Grading of Recommendation, Assessment, Development, and Evaluation system. Included studies will be assessed by online application, GRADEpro (https://gradepro.org/). Outcomes will be graded into one of 4 levels—high, moderate, low, and very low.^[21]^

### 2.6 Protocol development and potential amendments

We plan to strictly observe this protocol to complete this systematic review and meta-analysis. However, if the protocol is changed, the information will be described in the final review.

## 3 Discussion

The high morbidity and mortality of CSAP associated with anxiety and depression is of concern. Moreover, some studies have found that anxiety and depression often accompany CSAP and can exacerbate the condition of CSAP. However, there is no intervention that is both effective and side-effect free. According to theories of traditional Chinese medicine (TCM), the heart governs mind. In other words, CSAP, anxiety, and depression have close correlations with the heart. As a major part of TCM, acupuncture may have the potential to manage the symptoms of CSAP, anxiety, and depression simultaneously. Nowadays, acupuncture is receiving increasing popularity worldwide, because its efficacy is recognized by more clinicians and has the advantage of less side effects. Previous studies have indicated that acupuncture can effectively reduce blood pressure, diminish cardiac oxygen demand, arrhythmias, relieve the symptoms of myocardial ischemia, improve cardiac function, and prevent the recurrence of angina pectoris.^[17,22–25]^ Meanwhile, acupuncture can rapidly increase the release of endogenous opioid peptides or increase nocturnal melatonin secretion to relieve anxiety and depression.^[26,27]^ Previous studies have shown that acupuncture treat angina pectoris, relieve anxiety and depression by regulating the central nervous system.^[28–30]^ However, it remains unclear whether acupuncture can treat both diseases at the same time.

The vast majority of previous systematic reviews and meta-analysis have evaluated the curative effect of acupuncture on CSAP, but they overestimated the high incidence of CSAP-related anxiety and depression. Therefore, we first performed the meta-analysis, which may provide reliable and convincing evidence for the efficacy of acupuncture for treating CSAP-associated anxiety and depression. Conclusions from this review may benefit patients with CSAP-associated anxiety and depression, as well as clinicians and researchers. If this protocol needs to be amended, we will provide the date of each modification with statement of the changes and the corresponding reasons.

## Author contributions

JF conceived the study and provided general guidance to the drafting of the protocol. MT, JY, and BL did a preliminary investigation. YJ, JY, and MT drafted the protocol. YJ, JY, and MT designed the search strategy. MT and JY drafted the manuscript. JF and YJ reviewed and revised the manuscript. All authors have read and approved the final version of the manuscript.
